# Daily Association Between Sleep and Stressors: Role of Personality Traits

**DOI:** 10.1093/geroni/igaa057.1375

**Published:** 2020-12-16

**Authors:** Angelina Venetto, Taylor Vigoureux, Soomi Lee

**Affiliations:** University of South Florida, Tampa, Florida, United States

## Abstract

Poor sleep is associated with more stress across adult populations. The sleep—stress relationship is particularly important in nurses who are vulnerable to daily work-related stressors and poor sleep. Nurses with certain personality traits may be more vulnerable, however, the role that personality plays in the sleep-stress relationship has not previously been examined with lack of research in nurses. We examined how personality moderated the association between sleep characteristics and the perception of daily stressors in nurses. Participants were 61 oncology nurses who responded to a background survey that included a personality measure and completed 14 days of ecological momentary assessments. Each morning, participants reported sleep characteristics (i.e., perceived sleep sufficiency, sleep duration). Three times daily, participants reported their stressor experiences. We used multilevel models adjusting for sociodemographic characteristics, work shift, and work day. Results showed that on average across 2 weeks, participants with higher sleep sufficiency (β=-21.06, p<.05) and longer sleep duration (β=-11.80, p<.05) reported lower stressor severity. Agreeableness moderated the sleep duration—stressor severity association (β=25.07, p<.01), such that longer sleep duration was associated with lower stressor severity for participants with lower agreeableness (β =-17.39, p<.01), but not those with higher agreeableness (β=5.66, p>.05). These findings indicate that the protective nature of longer sleep duration on stressful experiences may not occur in nurses high in agreeableness. Nurses high in agreeableness may take on more responsibilities, exposing themselves to more daily stress. Thus, nurses who are high in agreeableness may be a good target population for stress-reduction interventions.

